# Evidence of artificial-refuge avoidance in the Ladder Snake (*Zamenis scalaris*) and the Montpellier Snake (*Malpolon monspessulanus*)

**DOI:** 10.3897/BDJ.13.e170781

**Published:** 2025-10-13

**Authors:** Grégory Deso, Gonzalo del Barrio

**Affiliations:** 1 Herpetologist AHPAM (Association Herpétologique de Provence Alpes Méditerranée), Orange, France Herpetologist AHPAM (Association Herpétologique de Provence Alpes Méditerranée) Orange France; 2 Clinique vétérinaire Sainte-Anne, Sorgues, France Clinique vétérinaire Sainte-Anne Sorgues France

**Keywords:** reptile conservation, avoidance measures, artificial refuges, radio-tracking, Mediterranean snakes, *Zamenis scalaris*, *Malpolon monspessulanus*

## Abstract

Artificial refuges are increasingly used as mitigation tools to provide alternative shelters for reptiles affected by habitat disturbance, but their short-term effectiveness remains poorly assessed, particularly in Mediterranean snakes. During construction works in southern France, six snakes (3 *Zamenis scalaris* and 3 *Malpolon monspessulanus*) were captured in the impacted area, fitted with VHF transmitters and released directly into artificial shelters within large-mesh fenced enclosures that allowed snakes and other fauna to move in and out freely. These enclosures preserved fragments of natural habitat from heavy construction impacts, while remaining connected to surrounding habitats. Radio-tracking (September 2024 – May 2025) revealed that all individuals left the artificial refuges immediately after release and never reused them, even during or after hibernation. Instead, they consistently selected nearby natural shelters, mainly sun-exposed rodent burrows. During this period, home ranges (MCP) varied between 2.86–8.79 ha in Z. scalaris and 0.54–3.78 ha in M. monspessulanus, a seasonal pattern that does not reflect their annual space use. This study provides the first experimental evidence that, in these two species, artificial refuges are ineffective in the short term, even when used as initial re-introduction sites. In contrast, fenced enclosures proved effective as an immediate mitigation measure, whereas artificial shelters may require long-term ecological stabilisation before becoming functional.

## Introduction

With the increasing artificialisation of natural habitats, reptile avoidance measures have become a common practice to mitigate the direct impacts of development projects on biodiversity (Germano and Bishop 2009, IUCN/SSC 2013, Choquette et al. 2024). Artificial refuges are designed to provide alternative shelters for reptiles, particularly during hibernation, a critical phase of their life cycle. Based on ecological principles, these structures aim to reproduce the thermal and microclimatic conditions of natural refuges. Physiological constraints, such as tolerance to temperature and humidity, are known to strongly influence survival during hibernation (Costanzo 1989, Wu et al. 2025). The importance of underground shelters for reptile survival has been demonstrated in both snakes and lizards (Grillet et al. 2010, Bonnet et al. 2016). However, several studies suggest a gradual colonisation of artificial refuges, sometimes requiring years before ecological stabilisation is achieved (McKelvey 2024). In Mediterranean landscapes, where anthropogenic and agricultural pressures are particularly strong, the availability of suitable shelters is a key factor for the persistence of snake populations.

The Montpellier Snake (*Malpolon monspessulanus* (Hermann, 1804)) and the Ladder Snake (*Zamenis scalaris* (Schinz, 1822)) are two diurnal colubrids widely distributed in Mediterranean landscapes, but increasingly affected by habitat fragmentation and land-use change. These large snakes strongly depend on underground refuges for hibernation and predator avoidance. Yet, their ecology in human-modified environments remains poorly documented, particularly regarding their response to artificial refuges used as mitigation tools. Understanding their short-term behaviour towards these structures is therefore essential to assess the effectiveness of current conservation practices.

Here, we present the first short-term evaluation of artificial refuges in the Ladder Snake and the Montpellier Snake, based on enclosures established as avoidance measures during construction works in southern France. These enclosures were fenced with large-mesh wire, allowing reptiles and other small fauna to move in and out freely. Our aim is to determine whether these snakes immediately accept or reject such structures, to compare these results with the use of enclosures preserving parts of their habitat and to analyse the implications for short- and long-term conservation strategies.

## Material and methods

From September 2024 onwards, six snakes (3 *Zamenis scalaris* and 3 *Malpolon monspessulanus*) were captured in an area impacted by the development of an industrial estate (ZAC) in the Municipality of Entraigues-sur-la-Sorgue. The snakes were fitted with VHF radio transmitters (Holohil, model SB-2, 5 g), representing less than 5% of body mass for all individuals, including the smallest Ladder Snake (*Zamenis scalaris*, 207 g). Surgical implantation was performed in a veterinary clinic by a licensed veterinarian via an intra-coelomic approach under isoflurane anaesthesia, following the protocol described by del Barrio et al. In press for both species and in accordance with international guidelines for snake radiotelemetry. Individuals were subsequently tracked using an R-1000 receiver coupled with a three-element Yagi antenna. After a 24–48 h recovery period in a heated terrarium with access to water and food to promote healing, each individual was released directly into an artificial refuge located within an avoidance enclosure (“esclot”) fenced with large-mesh wire, allowing snakes to freely enter and exit. These enclosures were situated close to the original capture site and, thus, most likely within the natural home range of each snake (Fig. 3)

**Figure 1.**
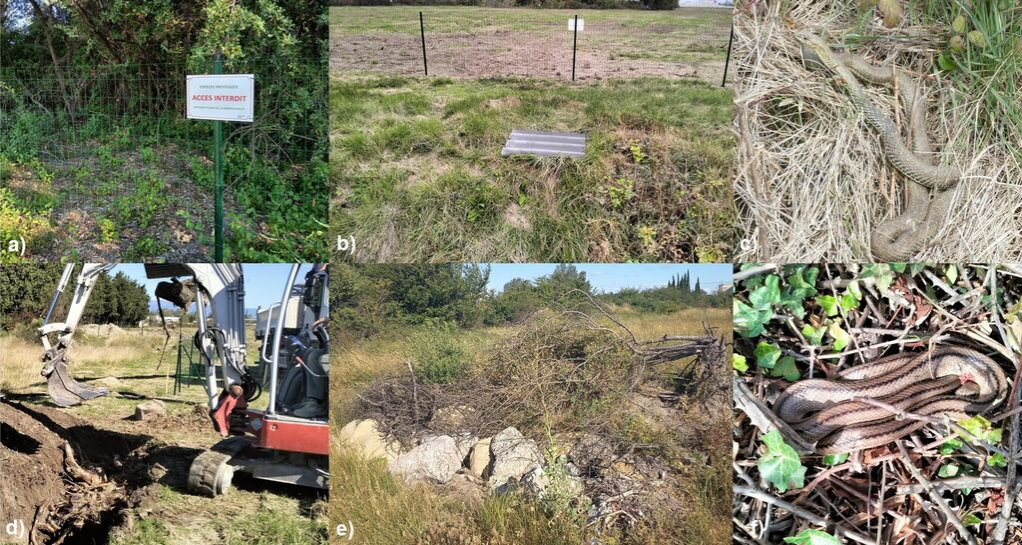
Examples of mitigation measures and snakes observed during the study in Entraigues-sur-la-Sorgue (Vaucluse, France): a) fenced avoidance enclosure with biodiversity protection sign; b) fibre-cement sheet used as a temporary refuge for surveys inside the enclosure; c) Montpellier Snake (*Malpolon monspessulanus*) in natural vegetation within the enclosure; d) mechanical excavation for the creation of an artificial hibernaculum; e) completed artificial refuge filled with branches, logs, stones and rocks; f) Ladder Snake (*Zamenis scalaris*) using an edge habitat inside the enclosures.

The avoidance enclosures covered a total of approximately 4 ha of natural habitats and included nearly 2,600 m of fencing. The mesh size was wide enough to allow the passage of small fauna (Fig. 1). The fencing was not designed to be snake-proof. In practice, these fences delimited patches of preserved natural habitats (“esclots”) within the construction site. Some areas were partially fenced, others entirely, but all remained connected to surrounding natural habitats outside the enclosures. The primary function of these fences was to limit the intrusion of heavy construction machinery (20-tonne excavators, vegetation shredders, tree-cutting machines), thereby preserving residual habitats. Thus, snakes could cross them to reach other natural habitats.

Artificial refuges measured 3 × 1.5 m, with a minimum depth of 1 m. They consisted of a pit filled at the base with plant substrate (logs, branches, leaves), covered with stones of varying sizes. This design was intended to mimic natural hibernation refuges, as frequently recommended by environmental consulting firms in a mitigation context. The lack of description of the technical characteristics and implementation costs of wildlife conservation measures is a major obstacle to their application and evaluation (Laurence et al. 2025). We therefore detail below the various costs of the structures and associated monitoring: total ≈ €70,000, including ~ €50,000 for the supply and installation of fencing; €1,900 for the creation of refuges (rental of an excavator, driver and herpetologist assistance); €1,000 for the delivery of materials by quarry operators; €1,000 in veterinary expenses (including the purchase of six Holohil transmitters); and €15,000 for monitoring (at a rate of 1 - 3 visits per week). Radio-tracking was carried out from 10 September 2024 to 30 May 2025 in order to document movements, refuge selection and hibernation behaviour. Each individual was located two to three times per week using an R-1000 receiver and a three-element Yagi antenna, resulting in 49 to 73 locations per snake (see Table 1). The accuracy of locations was estimated at < 5 m using a Redmi Note 5G smartphone with the Qfaune application (a spatial mapping tool derived from QGIS for mobile devices) and the data were subsequently exported in .xls format for analysis. Home ranges were calculated using 100% minimum convex polygons (MCP), a method widely used in herpetology. Spatial analyses were performed in R (version 4.3.2) using the adehabitatHR package. Fibre-cement sheets, known to be used by reptiles for thermoregulation while remaining protected from predators, were also installed to monitor herpetofaunal diversity within the enclosures (Lelièvre et al. 2010, Billy et al. 2024). A total of 25 sheets were distributed across the enclosures and preserved movement corridors and they were checked once per week throughout the monitoring period. We specify that these sheets were new models supplied by the Eternit France group, asbestos-free. All procedures were conducted in accordance with international guidelines on the ethical use of reptiles in research (ASIH/HL/SSAR et al. 2004). The study was carried out under the environmental permit, including a derogation for the protection of species, issued by the Prefecture of Vaucluse for the Natura Parc project in Entraigues-sur-la-Sorgue, following the deliberated opinion no. MRAe2024APPACA4/3581 of 25 January 2024 and the subsequent prefectoral decree (final authorisation delivered in 2024). Field tracking started on 10 September 2024.

## Results

**Figure 2.**
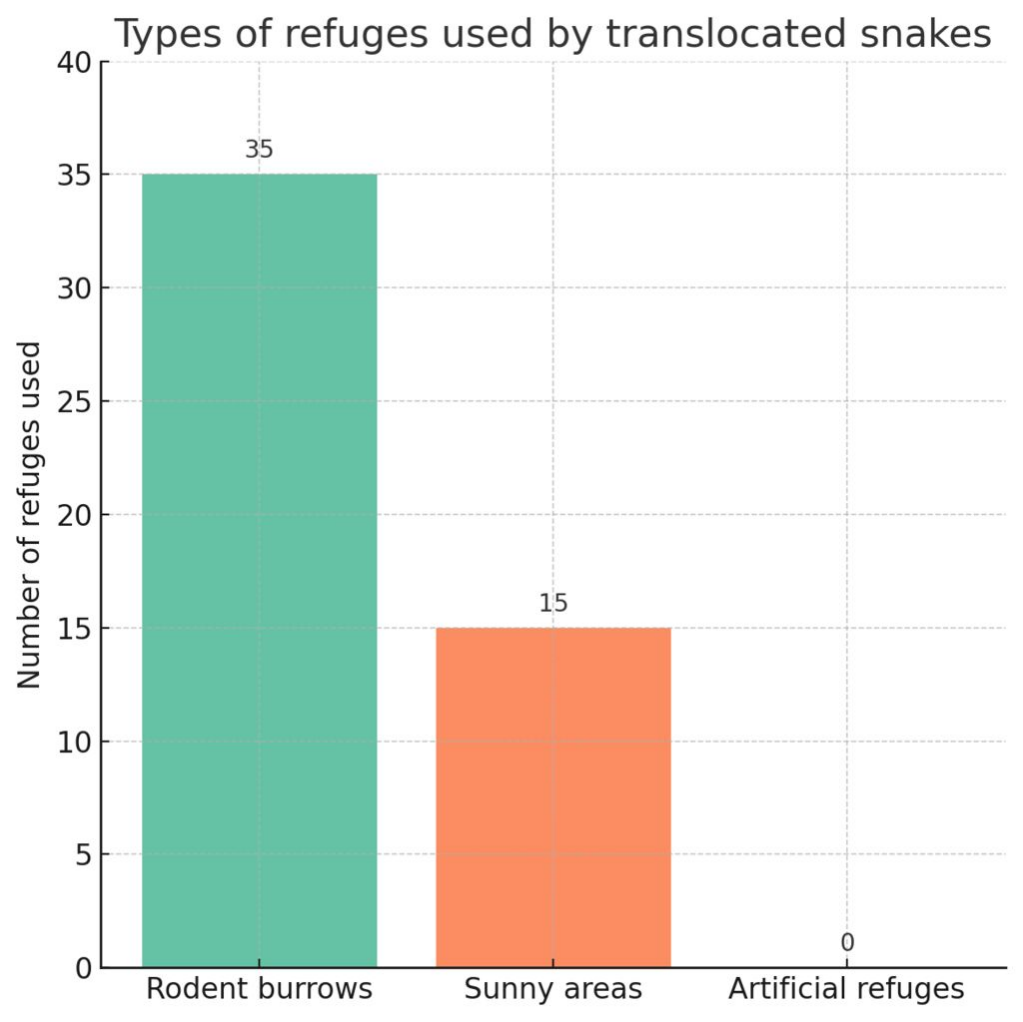
Types of refuges used by *Zamenis scalaris* and *Malpolon monspessulanus* during the monitoring period. Natural refuges (rodent burrows and sunny areas) were consistently selected, while artificial refuges were never used.

**Figure 3.**
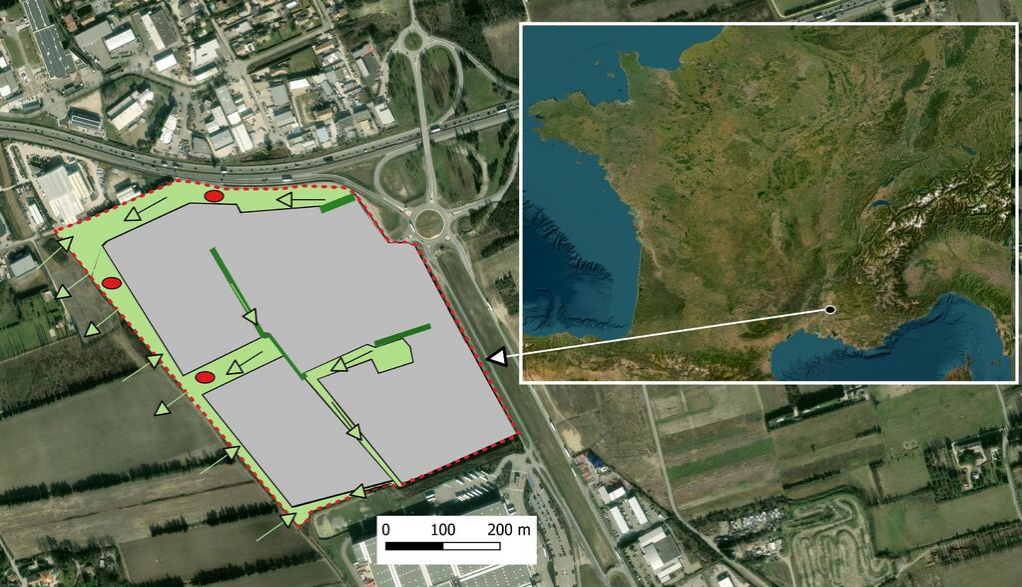
Study area at Entraigues-sur-la-Sorgue (Vaucluse, France) showing fenced enclosures with preserved habitat and home range allowing snakes to enter and exit (light green), unfenced corridors (dark green), experimental refuges (red circles), arrows indicating possible snake movements and areas under current or planned destruction (grey).

All snakes left the artificial refuges immediately after release and never re-used them during the eight months of monitoring, including for hibernation. In contrast, some individuals were re-observed in the same natural shelters used during their initial capture or subsequent veterinary releases during the monitoring period, confirming their fidelity to known natural shelters, whereas none ever returned to the artificial refuges. Instead, they exclusively selected natural refuges, mainly rodent burrows often highly exposed to sunlight. By “refuge,” we specifically refer to sites used as resting or hibernation shelters, as well as certain temporary refuges. However, some shelters occasionally used corresponded to simple thickets or shaded areas, allowing snakes to rest or to remain concealed while ambushing prey, but not suitable for hibernation. In Fig. 2, we therefore distinguish “sunny areas” as temporary shelters.

Each individual was regularly tracked, with a total of 73, 55 and 49 locations for the Montpellier Snake (*Malpolon monspessulanus*) and 70, 53 and 63 locations for the Ladder Snake (*Zamenis scalaris*) (range: 49–73), providing robust estimates of home ranges (Table 1).

All snakes survived the monitoring period. They remained inside the enclosures until the end of hibernation and, although they showed some activity with a few movements in autumn and very limited ones during winter, more extensive movements — including fence crossings to reach other natural habitats as well as returns to the enclosures — only began in March 2025.

Morphological characteristics, tracking effort and MCP values are summarised in Table 1, while the distribution of exploited refuges is illustrated in Fig. 2.

It is important to emphasise that these results reflect a pre-hibernation autumnal spatial use pattern and, therefore, do not represent annual space use. In this context, Ladder Snakes showed larger MCPs (2.86–8.79 ha), while Montpellier Snakes occupied smaller home ranges (0.54–3.78 ha) as hibernation approached. Interindividual differences were observed: some Ladder Snakes rapidly explored large areas in search of natural refuges sometimes located at a distance, whereas other individuals (particularly some Montpellier Snakes) remained restricted to sectors closer to the release area, exploiting numerous nearby natural refuges that they may have been familiar with prior to the construction phase. Such differences may reflect sex-related, size-related or individual variation. Sexual dimorphism has been reported in both species (e.g. tail proportions in *Z. scalaris*, larger body size in male *M. monspessulanus*; Feriche et al. (2008), Pleguezuelos 2019, Pleguezuelos 2021). However, previous studies found no significant sex differences in home range size in *M. monspessulanus* (Monrós 1997) or *Z. scalaris* (Blázquez 1993, Blázquez 1995, Martinez-Freiria et al. 2018), suggesting that much of the observed variability may reflect individual behaviour differences (Skinner et al. 2022). Given our small sample size, these patterns should be interpreted with caution.

## Discussion and conclusions

This study provides the first experimental evidence, in the Ladder Snake (*Zamenis scalaris*) and the Montpellier Snake (*Malpolon monspessulanus*), that artificial refuges are not immediately attractive, even when snakes are directly released into them. Instead, they exclusively used natural shelters available inside the enclosures, just behind the fences at the interface between the construction site and the surrounding wild habitats, mainly rodent burrows often strongly exposed to sunlight. They also used shelters in unfenced movement corridors that were temporarily preserved at the interface. All these shelters, whether behind the fences or within the movement corridors, most likely corresponded to familiar refuges already known before the construction works. Convergent results showing low short-term adoption of artificial hibernacula in North American snakes have also been reported (McKelvey 2024). Studies have shown that snakes often abandon release shelters shortly after being freed, actively exploring multiple shelters within their territories, but they may later return, as shelter-site fidelity has also been documented (Reinert and Rupert 1999, Plummer and Mills 2000, Sullivan et al. 2014). Evidence of individual variability in habitat use has also been reported in snakes (Row and Blouin-Demers 2006). In our study, several snakes were also re-observed in the same natural shelters used during their initial capture or during sanitary releases carried out during veterinary checks in the course of monitoring, confirming that they do not systematically avoid release sites throughout the monitoring period. In contrast, none ever returned to the artificial refuges, which clearly indicates a specific lack of attraction to these structures rather than a generalised escape response. The lack of structural complexity and less favourable microclimatic conditions probably explain this avoidance, but other factors, such as the absence of chemical cues (e.g. rodent or snake scent), may also play a role in the rejection of artificial refuges.

The avoidance enclosures proved to be more effective: snakes remained in secured areas throughout hibernation while displaying natural refuge selection behaviour (Germano and Bishop 2009). However, their persistence inside the enclosures or just behind the fences in natural areas adjacent to the construction site cannot be attributed solely to the fences. These zones also contained functional habitats preserved from the works that met their ecological requirements. The snakes’ continued use of these habitats over several months demonstrates their familiarity with numerous refuges and potential food sources. This confirms that the maintenance of natural microhabitats is essential for reptile persistence (Lecq et al. 2015, [Bibr B13444439][Bibr B13444550],Lecq et al. 2018). In grass snakes (*Natrix* spp.), several studies have highlighted the selection of thermally favourable habitats and strong variability in home range size depending on local conditions (Madsen 1984, Wisler et al. 2008), although published data specifically on hibernaculum choice remain limited. These parallels confirm that microclimatic stability is a key factor in refuge selection in many snakes and probably explains the short-term avoidance of artificial structures observed here.

In the context of mitigation measures during construction works, fenced enclosures should be prioritised to protect reptiles in the short term, while artificial refuges should be implemented experimentally. Although ineffective in the immediate term, several lines of evidence suggest they may become functional over time and gradually compensate for the loss of natural shelters. For example, Bonnet and Deso (2024) presented an artificial refuge monitored by video in an intensive agricultural landscape, spontaneously colonised by Montpellier Snakes about one year after its creation. Long-term monitoring is, therefore, essential to fully validate their role in conservation strategies. Baiting experiments have already been used to detect snakes by inducing them to leave their shelters (Deso et al. 2022, Deso and Bonnet 2023). In the same perspective, olfactory enrichment could be experimentally tested to enhance the attractiveness of newly-created refuges, for example, by adding rodent bedding or conspecific scent (Lah et al. 2025), thereby increasing their potential as protective shelters.

It is also noteworthy that the snakes did not choose to hibernate much further away from the construction site. Other wild habitats were available a few hundred metres away, but these may already have been occupied by other snakes.

Finally, the limitations of this study should be acknowledged: the small sample size (six individuals), the absence of information on home ranges prior to capture and the short monitoring period restrict the generalisation of the results. Nevertheless, our findings represent an important first step towards understanding the short-term response of Mediterranean snakes to artificial refuges and highlight the need to associate them with the preservation of natural habitats, so that snakes can maintain their spatial landmarks through the persistence of natural microhabitats within development projects.

## Figures and Tables

**Table 1.** Physical characteristics, tracking effort and Minimum Convex Polygon (MCP) home range size for each individual.

**Species**	**Sex**	**TL (cm)**	**SVL (cm)**	**Mass (g)**	**Tracking period**	**Tracking points**	**MCP (ha)**
*Zamenis scalaris* 1	Male	130	117	700	10/09/24–21/05/25	70	2.86
*Zamenis scalaris* 2	Female	102	92.5	283	17/09/24–22/04/25	53	8.79
*Zamenis scalaris* 3	Male	94	78.5	207	11/10/24–30/05/25	63	4.86
*Malpolon monspessulanus* 1	Male	126	98.5	394	06/09/24–30/05/25	73	3.78
*Malpolon monspessulanus* 2	Female	102	77	202	30/10/24–23/05/25	55	1.65
*Malpolon monspessulanus* 3	Female	131.5	101	399	13/11/24–23/05/25	49	0.54
